# Tétraparésie révélant un adénome de Conn chez une femme enceinte

**DOI:** 10.11604/pamj.2016.25.24.8245

**Published:** 2016-09-27

**Authors:** Naoufal Assoufi, Nessrine Bahadi, Nawal EL Omri, Youssef Sekkach, Taoufiq Ameziane, Driss Ghafir

**Affiliations:** 1 Service de Médecine Interne, Hôpital Militaire d’Instruction Mohammed V, Faculté de Médecine et de Pharmacie, Université Mohammed V, Rabat, Maroc

**Keywords:** Adénome de Conn, tétraparésie, rhabdomyolyse, hypokaliémie, Conn adenoma, tetraparesis, pregnancy, hypokalemia, rhabdomyolysis

## Abstract

Nous rapportons le cas d’un adénome de Conn révélé par une tétraparésie chez une femme de 33 ans, enceinte à 16 semaines d’aménorhées. La patiente a présenté une tension artérielle à 147/87 mmHg qui paraissait normale haute avec une hypokaliémie à 1,1 mmo/l. Le diagnostic a été confirmé par le dosage hormonal qui a montré une élévation de l’aldostérone plasmatique et une baisse de l’activité rénine plasmatique. L’imagerie par résonance magnétique a mis en évidence un nodule surrénalien gauche de 1,5 centimètre de diamètre pouvant être compatible avec un adénome surrénalien. Une surrénalectomie gauche a été pratiquée avec des suites opératoires simples, normalisation de la kaliémie et de la tension artérielle.

## Abstract (en)

We report the case of Conn adenoma revealed by tetraparesis in a 33-year old pregnant woman at the 16^th^week of amenorrhea. The patient had a blood pressure of 147/87 mmHg, which was considered high-normal, hypokalemia at 1.1 mmol/l. The diagnosis was confirmed by hormone dosage which showed elevated plasma aldosterone levels and decreased plasma renin activity. MRI showed a left adrenal nodule, 1.5 cm in diameter, compatible with adrenal adenoma. Left adrenalectomy was performed with simple postoperative course and normalization of kalemia and blood pressure.

## Introduction

L’hyperaldostéronisme primaire constitue une cause rare d’hypertension artérielle (HTA). Il est classiquement défini par une production excessive d’aldostérone, responsable d’une hypervolémie avec hypertension artérielle et une hypokaliémie d’origine rénale. Les manifestations cliniques, liées à l’hypokaliémie, sont le plus souvent modérées et potentiellement réversible, les formes neuromusculaires sévères ne le sont qu’exceptionnellement. Nous rapportons le cas d’une tétraparésie avec rhabdomyolyse secondaire à une hypokaliémie chez une femme enceinte présentant un hyperaldostéronisme primaire.

## Patient et observation

**Figure 1:**
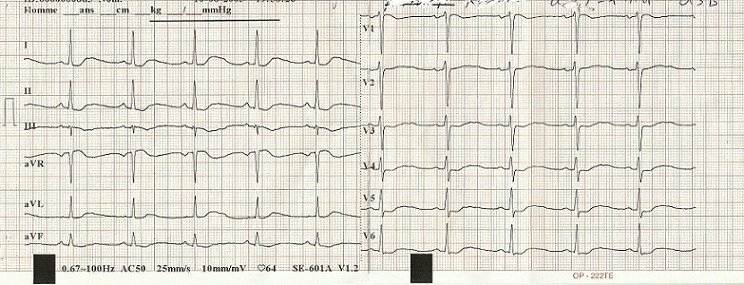
Électrocardiogramme montrant des troubles de la repolarisation

**Figure 2:**
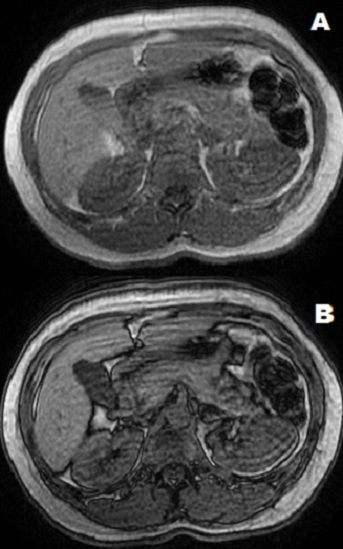
En phase: nodule surrénalien en iso signal T1 (A) et T2 (B) avec une chute partielle du signal en opposition de phase témoignant d’une composante graisseuse intra lésionnelle

Mme E.K, âgée de 33 ans, enceinte à 16 semaines d’aménorrhée, sans antécédents particuliers, admise pour une impotence fonctionnelle des quatre membres et algies diffuses avec notion d’urines foncées, évoluant depuis trois mois dans un contexte d’asthénie et d’apyrexie. L’examen général trouvait une patiente consciente, bien orientée dans le temps et dans l’espace, pesant 73 Kg pour une taille de 1.61 M, avec une pression artérielle (PA) à 148/87 mmHg. L’examen neurologique montrait un signe de tabouret positif et une diminution bilatérale de la force segmentaire et globale des quatre membres sans signes pyramidaux ni troubles sensitifs ou troubles vésico-sphinctériens. L’examen des paires crâniennes était normal. L’auscultation cardiaque était normale. L’abdomen était souple sans masse palpable. Le bilan biologique mettait en évidence une hypokaliémie à 1,1 mmol/l (normale: 3,5 à 4,5 mmol/l), avec une kaliurèse élevée à 64 mmol/24h, une élévation du taux des enzymes musculaires (créatine phosphokinase (CPK) = 7722 UI/l et lactate déshydrogénase (LDH) = 313 UI/l) et des transaminases avec des ASAT à 116 IU/l et des ALAT à 100 UI/L.la natrémie était normale à 140 mmol/l ainsi que la natriurèse qui était à 108 mmol/l.la créatininémie était normale à 6 mg/l. Le bilan hormonal montrait un hyperaldostéronisme avec un taux d’aldostérone à 4275 pmol/L en position couchée et à 4838 pmol/L en position debout (normale < 440 pmol/L en position couchée et 110 à 880 pmol/L en position debout). L’activité rénine plasmatique etait diminuée à 1,5 mUI/l en position debout pour une normale comprise entre 4,4 et 46,1 mUI/Let à 1,4mUI/l en position couchée pour une normale comprise entre 2,8 et 39,9 mUI/L. Le reste du bilan hormonal notamment le bilan thyroidien était normale avec une TSH à 2,96 mUI/l, une T4 à 13,98 pmol/l L’électrocardiogramme (Figure 1), réalisé au repos avant la correction de la kaliémie, inscrivait un rythme cardiaque à 62 b/min, un affaissement du segment ST et un aplatissement des ondes T. Devant l’hypokaliémie sévère et symptomatique, la patiente bénéficiait aussitôt d’une recharge potassique par une solution de chlorure de potassium, à travers une voie veineuse centrale, sous contrôle électrocardioscopique. Le diagnostic d’hyperaldostéronisme a été posé et la patiente mise sous spironolactone 75 mg (un comprimé deux fois par jour) associé au potassium. Les contrôles biologiques montraient une kaliémie qui se normalisait progressivement et l’évolution neurologique était favorable, avec disparition complète de la tétraparésie. L’IRM abdominale réalisée avait mis en évidence un nodule de la partie inferieure du corps surrénalien gauche de 15mm x13mm de diamètre pouvant être compatible avec un adénome surrénalien (Figure 2). L’évolution été marquée par la survenue d’une thrombose veineuse profonde sur cathéter fémoral, nous obligeant à différer l’intervention chirurgicale. A 34 semaines d’aménorrhée la patiente a été admise pour pré éclampsie et a subit une césarienne en urgence. Deux mois plus tard, la patiente a bénéficié d’une surrénalectomie gauche avec des suites opératoires simples, une normalisation de la kaliémie et de la pression artérielle.

## Discussion

L’adénome de Conn est une pathologie rare puisque elle ne représente que 1% des incidentalomes surrénaliens [1] et moins de 1% des patients hypertendus [2]. C’est une tumeur bénigne de la corticosurrénale avec une hypersécrétion isolée d’aldostérone [3]. Les manifestations sont le plus souvent une hypertension artérielle associée à une hypokaliémie. Les manifestations neuromusculaires des hypokaliémies causées par ce syndrome sont variées et non spécifiques. Elles peuvent aller de la simple fatigabilité à de véritables paralysies musculaires. Si la notion de paralysie secondaire à une dyskaliémie est connue, une tétraparésie associée à une rhabdomyolyse par hypokaliémie profonde observée dans le syndrome de Conn est une situation rarement décrite [4]. La pathogénie des paralysies musculaires survenant au décours d’une déplétion potassique rapide se résume en une augmentation de la différence de concentration transmembranaire en potassium ce qui favorise ainsi sa diffusion passive vers le milieu extracellulaire. Cela entraine un potentiel de repos transmembranaire plus important avec une membrane cellulaire hyperpolarisée et des cellules moins excitables [5]. L’ascension des enzymes musculaires (CPK, LDH) serait due à une altération des transports ioniques, à des déficits enzymatiques fonctionnels, à des anomalies de la synthèse de stockage de glycogne et à une inadaptation du flux sanguin musculaire à l’effort.

Le diagnostic d’hyperaldostéronisme primaire devant un tableau de tétraparésie peut se poser avec une lésion médullaire cervicale post-traumatique. Mais l’absence de notion de traumatisme permettait d’écarter cette hypothèse. Elle peut être secondaire à une atteinte médullaire (disque intervertébral, tumeur, lésions vasculaires), à la sclérose en plaques, à une infection ou à un abcès de la moelle épinière, et à une malformation congénitale [6, 7]. Dans ces cas, la paraplégie est habituellement associée à des troubles vésicosphinctériens et génito-sexuels. L’existence d’une hypokaliémie pouvait également faire évoquer la paralysie périodique hypokaliémique primitive qui est une affection autosomique dominante rare. Dans notre cas, en tenant en compte qu'il ya une tendance à l'hypotension artérielle dans le cadre des modifications physiologiques au cours du premier trimestre de la grossesse [8], l’association d'une hypertension artérielle qui paraissait normale haute à une hypokaliémie profonde était en faveur d'un syndrome de conn.la confirmation était faite par le dosage hormonal et l'IRM. La prise en charge de la tétraparésie et de la rhabdomyolyse est basée sur la correction progressive du poul potassique, permettant une restauration d'une activité cellulaire normale.la réduction de l'hypersécrétion de l'aldostérone ne se conçoit que par une ablation chirurgicale et la surrénalectomie laparoscopique est aujourd'hui la technique indiquée en première intention [9]. L’intervention chirurgicale normalise la kaliémie dans tous les cas et une guérison de l'hypertension artérielle est observée dans 50% des cas. Le début récent de l'hypertension artérielle par rapport à la chirurgie et la bonne réponse préopératoire à la spironolactone sont considérés comme des facteurs de pronostic d'une guérison [9].

## Conclusion

Le diagnostic de la tétraparésie consécutive à des hypokaliémies de syndrome de conn doit être évoqué en cas de contexte évocateur (HTA et hypokaliémie). Une attention particulière est de rigueur chez la femme enceinte où il faut avoir le réflexe de tenir en compte les variations hémodynamiques physiologiques surtout au cours du premier trimestre de la grossesse où il y´a une tendance à l´hypotension artérielle. Après confirmation, une recharge potassique prudente permet une disparition complète des troubles neuromusculaires. La surrénalectomie conduit à la normokaliémie et à un meilleur contrôle de l´hypertension artérielle.
